# Premature Rupture of Fetal Membranes: A Narrative Review Integrating Current Evidence and International Guidelines for Optimal Care

**Published:** 2026-04-13

**Authors:** Malarchy E. Nwankwo, Samuel N. Ugadu, Arinze C. Ikeotuonye, Richard O. Egeonu, George Uchenna Eleje, Betrand Obi Nwosu, Emmanuel I. Ogumu, Chijioke A. Ugochukwu, Chigozie Geoffrey Okafor, Chukwunwendu Aloysius Okeke, Ahizechukwu Chigoziem Eke

**Affiliations:** 1 Department of Obstetrics and Gynaecology, Nnamdi Azikiwe University Awka, Anambra State, Nigeria; 2 Department of Obstetrics and Gynaecology, Nnamdi Azikiwe University Teaching Hospital, Nnewi, Anambra State, Nigeria; 3 Department of Obstetrics and Gynaecology, Barking, Havering and Redbridge University Hospitals NHS, Romford, London; 4 Division of Maternal Fetal Medicine, Department of Gynecology and Obstetrics, Johns Hopkins University School of Medicine, Baltimore, Maryland, USA

**Keywords:** premature rupture of membranes, preterm premature rupture of membranes, preterm labour, preterm delivery, chorioamnionitis, cytokines

## Abstract

Premature rupture of fetal membranes (PROM) is a significant obstetric complication associated with preterm birth and increased maternal and neonatal morbidity and mortality, particularly in resource-limited settings. It is a multifactorial condition resulting from complex interactions between infectious, inflammatory, mechanical, and biochemical processes that weaken the chorioamniotic membranes. This narrative review synthesises current evidence and international clinical guidelines on the diagnosis and management of PROM. It examines the epidemiology, risk factors, and underlying pathophysiology, with emphasis on the roles of infection, inflammatory mediators, and matrix metalloproteinases. Diagnostic strategies are reviewed, including clinical assessment, conventional bedside tests, and emerging biomarker-based approaches such as placental alpha-microglobulin-1 and insulin-like growth factor binding protein-1. Evidence-based management approaches are discussed, including prophylactic antibiotics, antenatal corticosteroids, magnesium sulphate for fetal neuroprotection, selective use of tocolysis, and gestational age-specific timing of delivery. Emerging areas, including outpatient management, prevention strategies, amnioinfusion in preterm PROM, and considerations for cerclage retention and breech presentation, are also addressed. In conclusion, this review provides a comprehensive and up-to-date synthesis of current knowledge on PROM to inform clinical practice. Improved risk stratification, timely diagnosis, and adherence to evidence-based management remain essential to optimise maternal and neonatal outcomes.

## Introduction

Prelabour rupture of membranes (PROM) refers to rupture of the fetal membranes prior to the onset of uterine contractions and the establishment of labour[1]. Depending on the gestational age at which membrane rupture occurs, PROM is further classified into distinct clinical categories. When rupture occurs before the age of fetal viability, defined in many low-resource settings as 28 weeks of gestation, it is referred to as previable PROM. Rupture that occurs before 37 completed weeks of gestation is termed preterm PROM (PPROM), whereas term PROM refers to rupture of the membranes occurring at or beyond 37 weeks of gestation [1,2].

PROM represents a relatively common obstetric complication, affecting approximately 8–10% of all pregnancies worldwide [3]. The incidence of previable PROM is comparatively low, occurring in less than 1% of pregnancies, while PPROM complicates about 2–3% of pregnancies and accounts for nearly one-third of all preterm births (PTB).[1,2] PROM occurring at term constitutes the majority of cases, affecting approximately 8% of pregnancies. Overall, rupture of membranes occurs at term in nearly 80% of PROM cases. Following rupture at term, spontaneous labour typically ensues within a short period; approximately 60% of women go into labour within 24 hours, and up to 95% begin labour within 72 hours of membrane rupture.[4]

**Figure 1:**
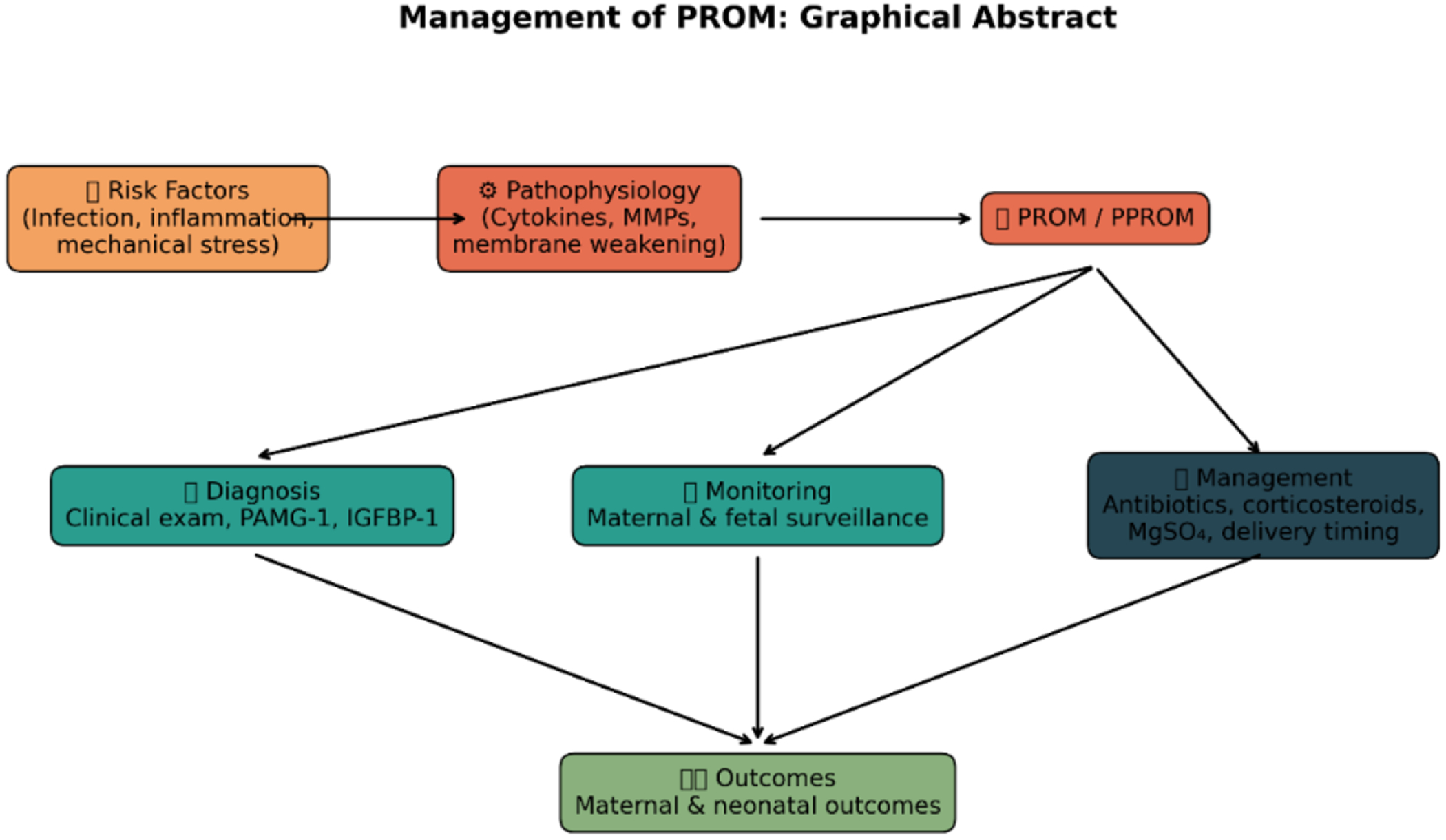
Schematic management of PROM

Data from local clinical settings also highlight the burden of PROM. In a five-year retrospective review involving 3,513 deliveries at the Nnamdi Azikiwe University Teaching Hospital (NAUTH), Nigeria, Eleje et al. reported an incidence of 2.4% for term PROM [5]. These findings emphasize the continued relevance of PROM as a significant obstetric condition that contributes to both maternal and neonatal morbidity (Figure 1).

This narrative review examines the epidemiology, risk factors, and aetiopathogenesis of premature rupture of fetal membranes (PROM), with particular emphasis on the roles of infection, inflammatory cytokines, and matrix metalloproteinases in membrane weakening and rupture. It further evaluates contemporary diagnostic approaches, including clinical assessment, conventional bedside tests, and emerging biomarker-based methods such as placental alpha-microglobulin-1 and insulin-like growth factor binding protein-1. Finally, the review synthesises current evidence and international clinical guidelines to inform the optimal management of PROM.

### Search strategy

A comprehensive and systematic literature search was conducted to identify relevant studies, guidelines, and evidence relating to the management of premature rupture of fetal membranes. The search process was guided by a structured query formulated as “Recent guidelines for management of premature rupture of fetal membranes.” This query was designed to capture the most up-to-date and clinically relevant publications addressing diagnosis, risk factors, complications, and management strategies for PROM.

Multiple electronic databases and information sources were explored to ensure broad coverage of the available literature. These included PubMed, Google Scholar, ResearchGate, Scopus, Web of Science, the Cochrane Library, and general Google searches, as well as relevant clinical protocols and international guideline documents. A wide range of keywords and search phrases were used either singly or in combination. These included premature rupture of fetal membranes, preterm premature rupture of fetal membranes, term premature rupture of fetal membranes, previable premature rupture of fetal membranes, chorioamnionitis, preterm labor, preterm delivery, inflammatory cytokines in labor, recent evidence for diagnosing premature rupture of fetal membranes, complications of premature rupture of fetal membranes, and management of premature rupture of fetal membranes.

Following the initial search, older publications with limited relevance to current clinical practice were excluded. A total of 123 publications were initially identified. After screening for relevance and methodological suitability, 92 publications were selected for detailed review and citation in this narrative review. Selection of the final references was based on their relevance to the research objectives, contribution to contemporary understanding of PROM, and applicability to clinical practice.

### Risk factors for PROM

A previous history of PROM represents the most significant and consistently reported risk factor for recurrence in subsequent pregnancies.[6] Aris et al. documented a recurrence risk of 6.6% among women with a prior history of PROM.[7] Evidence from a large prospective observational study conducted by the National Institute of Child Health and Human Development Maternal-Fetal Medicine Units Research Network further supports this association. In that study, 13.5% of women with a history of preterm birth resulting from PROM experienced PROM in a subsequent pregnancy, compared with 4.1% among women without a prior history of PROM, corresponding to a risk ratio (RR) of 3.3 (95% confidence interval [CI], 2.1–5.2).[8,9]

In addition to a prior history, several maternal, obstetric, and environmental factors have been associated with an increased risk of PROM. Genetic collagen vascular disorders, including Ehlers-Danlos syndrome, systemic lupus erythematosus, and Marfan syndrome, can compromise the structural integrity of the fetal membranes. Structural abnormalities of the uterus, cervical insufficiency, and previous cervical surgical procedures such as conization and loop electrosurgical excision procedure (LEEP) also contribute to membrane weakness and subsequent rupture [10,11].

Infectious and inflammatory conditions of the urogenital tract, as well as periodontal disease, have also been implicated in the pathogenesis of PROM. Other recognised obstetric risk factors include uterine overdistension resulting from polyhydramnios or multiple gestations, abdominal trauma, antepartum haemorrhage, and invasive prenatal diagnostic procedures such as amniocentesis, chorionic villus sampling, and cordocentesis. Environmental and socioeconomic factors further contribute to risk, including malnutrition, maternal anemia, low body mass index (BMI), cigarette smoking, and low socioeconomic status. [10,11]

More recently, Lin et al. conducted a systematic review and meta-analysis involving 21 studies and 18,174 women, which identified several additional factors significantly associated with PROM. These included low BMI, short interpregnancy interval (IPI <2 years), previous abortion, prior preterm birth, previous PROM, history of caesarean section, gestational hypertension, gestational diabetes mellitus, abnormal vaginal discharge, reproductive tract infections, fetal malpresentation, and increased abdominal pressure.[12] These findings underscore the multifactorial nature of PROM and the importance of identifying both modifiable and non-modifiable risk factors in clinical practice.

### Aetiopathogenesis of PROM

The aetiology of PROM is multifactorial, and the precise pathophysiologic mechanisms underlying membrane rupture remain incompletely understood. A central feature of PROM is disruption of the collagen framework of the chorioamniotic membranes, which compromises membrane strength and predisposes to rupture. Infectious and inflammatory processes play a major role in this collagen remodeling process.

Collagen vascular disorders can predispose women to PROM by altering the structural composition and mechanical integrity of the fetal membranes, which are largely composed of collagen. During intrauterine infection or inflammation, bacterial products and pro-inflammatory cytokines, particularly interleukin-1 beta (IL-1β) and tumour necrosis factor-alpha (TNF-α), stimulate the production of prostaglandins and increase the expression of matrix metalloproteinases (MMPs), also referred to as collagenases. These enzymes degrade the collagen matrix of the chorioamniotic membranes, leading to progressive weakening and eventual rupture.[11]

Biochemical studies have provided further insight into these mechanisms. Maymon et al. reported that PPROM is associated with increased amniotic fluid concentrations of MMP-1 and MMP-8 [13,14]. In addition, Maymon et al. and Athayde et al. demonstrated that women with microbial invasion of the amniotic cavity exhibit significantly higher concentrations of MMP-9 compared with women without microbial invasion[15,16].

Microbial infection has also been shown to play a significant role in PROM. Eleje et al. examined 210 endocervical swabs obtained from 105 women with PROM and 105 controls matched for age, parity, and gestational age [17]. Their findings revealed a significantly higher microbial culture positivity rate of 79.05% among cases compared with 6.67% among controls (p <0.05). Among the microorganisms identified, Streptococcus species were the most frequently isolated pathogens, occurring in 31.43% of cases compared with 4.81% of controls. Conversely, Candida albicans was significantly more prevalent in controls (27.62% vs 8.57%). Additionally, Gardnerella vaginalis and Trichomonas vaginalis were significantly more common among women with PPROM than among women with intact membranes.[17]

Periodontal disease has also been implicated in the pathogenesis of PROM. This condition represents a chronic inflammatory disorder of the supporting structures of the teeth and is dominated by gram-negative anaerobic bacteria. PROM may occur when oral bacteria gain access to the systemic circulation through bacterial invasion of the dental pulp following cavitation in untreated dental disease.[18]

Mechanical factors also contribute to membrane rupture. Mechanical stretching of the membranes, resulting from preterm uterine contractions or uterine overdistension in conditions such as polyhydramnios and multiple gestations, may weaken the membranes and predispose them to rupture. Additionally, fetal membranes may be iatrogenically ruptured during invasive procedures, including amniocentesis, chorionic villus sampling, and cordocentesis.[11]

### Diagnosis of PROM

In most cases, the diagnosis of PROM is made clinically. Approximately 90% of women present with a characteristic history of a sudden gush of fluid from the vagina followed by continuous leakage, which strongly suggests rupture of the membranes [19]. The diagnosis is confirmed when amniotic fluid is seen passing through the cervical os and/or when pooling of fluid is observed in the posterior vaginal fornix during sterile speculum examination [20]. Speculum examination also provides an opportunity to assess for cervical dilatation and effacement, cervicitis, and umbilical cord prolapse, and to obtain samples for microbiological studies where indicated. By contrast, digital vaginal examination should be avoided, unless the speculum findings suggest marked cervical dilatation, labour is established, or delivery appears imminent. This precaution is important because digital examination has been associated with increased risks of chorioamnionitis, neonatal infection, and shortened latency [3,20–22]. Since speculum assessment correlates well with digital findings in the evaluation of the cervix [23], it is generally sufficient in most clinical settings.

Despite the usefulness of history and examination, the diagnosis remains uncertain in about 10–20% of cases, necessitating the use of adjunctive tests. Traditional bedside tests include the nitrazine test and ferning test. A blue colour change on nitrazine paper and arborization on microscopy support the diagnosis of PROM; however, their diagnostic value is limited by false-positive results caused by semen, cervical mucus, blood, cervicitis, vaginitis, alkaline urine, and topical antiseptics [19,20]. The fetal fibronectin test is highly sensitive but lacks specificity for PROM. A negative result strongly suggests intact membranes, whereas a positive result does not establish the diagnosis with certainty. Accordingly, fetal fibronectin is more useful as a predictor of preterm birth than as a primary diagnostic test for PROM [24].

More invasive diagnostic procedures have also been described. In women with suspected PROM, amniocentesis followed by instillation of indigo carmine dye into the amniotic cavity may confirm rupture if blue-stained fluid leaks vaginally within 20–30 minutes. Although this method is diagnostically useful, it is invasive, costly, and associated with risks including iatrogenic PROM, placental abruption, infection, and miscarriage. The use of methylene blue is especially problematic because of its association with fetal hemolytic jaundice, hemolytic anaemia, hyperbilirubinaemia, and methemoglobinemia [25]. For these reasons, amnio-dye testing should not be used routinely in the diagnosis of PROM. Ultrasonography may demonstrate oligohydramnios, which can support the clinical impression, but it should not be used in isolation to establish the diagnosis. Its more important role lies in the assessment of fetal well-being, fetal presentation, placental location, estimated fetal weight, structural anomalies, and residual amniotic fluid volume [26].

When the diagnosis is equivocal, biochemical marker tests in vaginal secretions have emerged as valuable tools. These include placental alpha-microglobulin-1 (PAMG-1; AmniSure), insulin-like growth factor binding protein-1 (IGFBP-1; Actim PROM), and alpha-fetoprotein (AFP). Studies have shown these tests to be superior to conventional clinical methods in difficult cases [27,28]. Ibrahim et al. reported that the Amnioquick Duo+ test, which detects IGFBP-1 and AFP, had significantly greater diagnostic accuracy than both nitrazine and fern tests in identifying PROM [29]. In a comparative study, Eleje et al. found that Amnioquick Duo+ and PAMG-1 demonstrated similarly high diagnostic performance in equivocal cases, with a concordance rate of 97.0% [30]. In a meta-analysis, Palacio et al. showed that Actim PROM and AmniSure had comparable sensitivity (95.4% vs 96.7%) and negative predictive value (95.8% vs 96.7%), although AmniSure had higher specificity (98.3% vs 92.9%) and positive predictive value (98.3% vs 92.3%) [31]. In practical terms, PROM is unlikely when there is no clinical evidence of amniotic fluid leakage or pooling and PAMG-1 or IGFBP-1 testing is negative [32].

### Diagnosis of chorioamnionitis following PROM

Chorioamnionitis, whether diagnosed clinically or histologically, is a major complication of PROM and is reported in 30–80% of preterm births associated with PPROM [33]. Its diagnosis remains challenging because the clinical manifestations are often nonspecific and may overlap with other maternal or fetal conditions. The cardinal clinical signs include maternal fever (≥100.4°F), maternal tachycardia (>100 beats/min), fetal tachycardia (>160 beats/min), uterine tenderness, and purulent or foul-smelling vaginal discharge [3]. Diagnostic criteria vary across clinical practice. Some clinicians consider the presence of any two clinical features sufficient for diagnosis, while others require fever plus at least two additional signs, a stricter definition that appears to improve diagnostic accuracy [34,35]. Among these features, fever is the most sensitive, occurring in 95–100% of cases. Maternal tachycardia and fetal tachycardia show sensitivities of 50–80% and 40–70%, respectively, whereas uterine tenderness and offensive vaginal discharge are more subjective and are present in only 4–25% of cases [35].

Because clinical features alone lack specificity, laboratory evaluation is often used to support diagnosis. Leukocytosis (>12,000–15,000/mm3) is found in 70–90% of cases, but it is a weak standalone marker because elevated white cell counts may also occur in normal pregnancy, labour, and othe pathological states [35]. Similarly, cardiotocography and the feta biophysical profile perform poorly in predicting chorioamnionitis afte PROM [36]. Caloone et al. reported that C-reactive protein (CRP) was th best maternal serum marker for predicting chorioamnionitis in PROM [37] but Sabogal et al. found only modest diagnostic performance, with sensitivity of 68.7% and specificity of 77.1% [38]. Eleje et al. evaluated interleukin-6 (IL-6) in cervicovaginal secretions using the Chorioquick tes and demonstrated its potential clinical utility in detecting chorioamnioniti in women with PROM [39]. Nevertheless, a systematic review and meta-analysis by Etyang et al. concluded that there remains insufficient evidence to support routine use of CRP, procalcitonin, or IL-6 as reliable diagnosti markers for chorioamnionitis in PROM [40].

Taken together, the available evidence indicates that neither clinical sign nor laboratory markers are sufficiently sensitive or specific when used i isolation. Therefore, current recommendations favour a combined diagnosti approach, integrating clinical findings with relevant laboratory parameter when evaluating women with PROM for possible chorioamnionitis [3,32].

## Management of PROM

### Antibiotic prophylaxis following PROM

Administration of prophylactic antibiotics after PROM has been shown to reduce both maternal and neonatal morbidity. Documented benefits include lower rates of chorioamnionitis, neonatal infection, surfactant use, neonatal intensive care unit admission, and neonatal cerebral abnormalities, together with prolongation of the latency period [49]. Recommended regimens vary slightly across professional bodies. The National Institute for Health and Care Excellence (NICE) recommends oral erythromycin 250 mg four times daily for up to 10 days or until established labour, whichever occurs first [32]. This recommendation is largely based on the ORACLE I trial, which demonstrated more favourable maternal and neonatal outcomes with erythromycin than with co-amoxiclav alone or co-amoxiclav combined with erythromycin [50].

By contrast, the American College of Obstetricians and Gynaecologists (ACOG) recommends the regimen used in the National Institute of Child Health and Human Development Maternal–Fetal Medicine Units Network trial, consisting of intravenous ampicillin 2 g every 6 hours plus erythromycin 250 mg every 6 hours for 48 hours, followed by oral amoxicillin 250 mg every 8 hours and erythromycin base 333 mg every 8 hours for 5 days [51]. The Society of Obstetricians and Gynaecologists of Canada (SOGC) accepts either of these regimens [52]. Importantly, co-amoxiclav should be avoided because of its association with necrotizing enterocolitis in the neonate [50].

### Corticosteroid use in women with PROM

Antenatal corticosteroids remain a key component of care in women with PROM who are at risk of preterm birth. Their use reduces neonatal mortality and major neonatal morbidity, particularly intraventricular haemorrhage and respiratory distress syndrome, without increasing the risk of chorioamnionitis or neonatal infection [53]. The WHO Action Trial, which assessed the safety and effectiveness of antenatal glucocorticoids in low-resource settings, found that among women at risk of preterm birth, dexamethasone significantly reduced stillbirth and neonatal death without increasing maternal bacterial infection [54]. A Cochrane review of 12 trials involving 1,557 women and 1,661 infants found that dexamethasone and betamethasone had comparable efficacy [55]. In most of the head-to-head trials, two 12 mg intramuscular doses of betamethasone given 24 hours apart were compared with four 6 mg intramuscular doses of dexamethasone given 12 hours apart, and ACOG endorses either regimen [56].

A single course of antenatal corticosteroids is recommended for women with PROM between 24+0 and 33+6 weeks of gestation when delivery is considered likely within the next 7 days [20,32,56]. Although routine steroid use in the late preterm period (34+0 to 36+6 weeks) has traditionally been approached cautiously because of the risk of neonatal hypoglycaemia, findings from the Antenatal Late Preterm Steroids (ALPS) trial, a multicentre randomized study involving 2,831 women in 17 centres, showed that steroids given before anticipated late preterm delivery significantly reduced respiratory distress syndrome, stillbirth, and neonatal death within 72 hours of birth [57]. Since the optimal benefit of corticosteroids extends for approximately 7 days, a single repeat course may be considered in women at less than 34 weeks’ gestation if more than 7 days have passed since the initial course and there is a high likelihood of preterm birth within 48 hours [32]. More than two courses should not be administered, given the adverse effects of repeated exposure on fetal growth and neurodevelopment [53].

### Administration of magnesium sulphate in women with PROM

The fetal neuroprotective role of antenatal magnesium sulphate in women with PPROM is well established. A Cochrane review including five trials and 6,145 babies showed that magnesium sulphate administered to women at risk of preterm birth significantly reduced the likelihood of cerebral palsy and gross motor dysfunction [58]. Although different dosing schedules were used across the included trials, there is no clear evidence favouring one specific regimen [59]. Most guidelines therefore recommend using the minimum effective dose to reduce the risk of toxicity. The most widely recommended approach consists of a 4 g intravenous loading dose given slowly over 15–30 minutes, followed by a maintenance infusion of 1 g/hour until birth or for a maximum of 24 hours, whichever occurs first [32,60,61].

In low-resource settings where continuous intravenous infusion may not be feasible because of staffing or monitoring limitations, an alternative regimen may be used: 4 g intravenously over 20–30 minutes, followed by 5 g intramuscularly into each buttock, and then 5 g intramuscularly into alternate buttocks every 4 hours for 24 hours [3]. Regardless of the regimen, women receiving magnesium sulphate require close clinical monitoring. Pulse rate, blood pressure, respiratory rate, and deep tendon reflexes should be assessed at least every 4 hours [32,61]. Treatment should be stopped if signs of toxicity develop, including absent patellar reflexes, respiratory rate below 12/minute, a diastolic blood pressure drop greater than 15 mmHg below baseline, or urine output below 100 mL over 4 hours [3]. Calcium gluconate should be readily available as an antidote. Current guidance recommends magnesium sulphate for women with PROM at risk of preterm birth between 24+0 and 33+6 weeks of gestation [32,60,61]. There is no evidence supporting repeat dosing after a completed initial course [3].

### Use of tocolytics in women with PROM

The role of tocolysis in women with PPROM remains limited. A Cochrane review of eight trials involving 408 women found that although tocolytic therapy prolonged latency and reduced birth within 48 hours, it was also associated with a higher frequency of 5-minute Apgar score <7, increased need for neonatal ventilation, and a significantly increased risk of chorioamnionitis [62]. On this basis, routine tocolysis is not recommended in women with PPROM, as the maternal infectious risk outweighs the uncertain neonatal benefit [63].

That said, short-term tocolysis for up to 48 hours may be considered in selected women with PPROM at less than 34 weeks’ gestation, mainly to permit completion of antenatal corticosteroids and/or facilitate in-utero transfer to a facility with appropriate neonatal care [63,64]. When tocolysis is indicated, nifedipine or atosiban are preferred first-line agents because they offer similar efficacy and perinatal outcomes [64]. A commonly recommended nifedipine schedule is an initial dose of 10–30 mg, followed by 10–20 mg every 4–8 hours until contractions cease or for a maximum of 48 hours [65]. Betamimetics should be avoided because of their potentially severe cardiovascular adverse effects [66]. If one agent fails, a second drug from a different class may be used, but combination tocolysis is not recommended [65,66].

### Timing of delivery in women with PROM

#### Previable PROM (<28 weeks of gestation)

Previable PROM is associated with substantial maternal and neonatal risk. A recent systematic review and meta-analysis of 19 studies involving 1,640 singleton pregnancies showed that expectant management after previable or periviable PROM was associated with high rates of spontaneous abortion, fetal demise, maternal chorioamnionitis, endometritis, neonatal sepsis, and neonatal death [67]. Smaller observational studies by Beydoun et al., Sim et al., and Fernandes et al. reported similarly poor outcomes [34,68,69]. In view of these risks, women with previable PROM should receive clear counselling regarding the prognosis of expectant management and should be offered termination of pregnancy [20]. For women who choose expectant management, counselling should be repeated and comprehensive, antibiotic therapy should be given, and antenatal corticosteroids should be administered once the pregnancy reaches viability. Tocolysis is not recommended in this group [20].

### Preterm PROM (28+0 to 36+6 weeks of gestation)

Management of PPROM in the viable preterm period depends largely on gestational age and the balance between the hazards of prematurity and the risks of intrauterine infection. In a systematic review and meta-analysis of five randomized controlled trials including 488 women, Al-Mandeel et al. compared expectant management with immediate delivery between 28 and 34 weeks of gestation. Rates of maternal infection, respiratory distress syndrome, and neonatal sepsis were similar between the two groups; however, neonatal death and caesarean section were significantly more frequent in the immediate-delivery group [70]. These findings indicate that immediate birth between 28 and 34 weeks confers no added benefit and may worsen important maternal and neonatal outcomes. In uncomplicated PPROM during this gestational window, expectant management reduces prematurity-related morbidity, and ACOG recommends continuation of pregnancy until 34 weeks, provided no contraindication arises [20].

Historically, immediate delivery was advised for women with PPROM between 34+0 and 36+6 weeks [1,71]. More recent evidence has modified this approach. Current guidance supports offering expectant management until 37 weeks in the absence of contraindications to continuing the pregnancy [63]. This shift is based on the Cochrane review by Bond et al., which compared planned early birth with expectant management in women with PPROM before 37 weeks and found no reduction in neonatal sepsis with early delivery. Instead, early delivery was associated with increased risks of respiratory distress syndrome, need for ventilation, neonatal intensive care unit admission, neonatal mortality, endometritis, induction of labour, and caesarean birth [72]. Follow-up evidence on behavioural and neurodevelopmental outcomes at 2 years of age found no difference between infants delivered after expectant management and those delivered immediately following late PPROM [73]. Together, these data support a more conservative strategy in stable women.

### PROM at term

At term, the clinical context differs because the fetus is mature and the major concern shifts toward prevention of ascending infection. Approximately 60% of women with term PROM enter spontaneous labour within 24 hours, and 95% do so within 72 hours [4]. A meta-analysis by Mozurkewich and Wolf, which included 23 studies and 7,493 women, found no difference in caesarean delivery or neonatal infection rates between immediate induction and conservative management, but showed lower rates of chorioamnionitis and endometritis with induction [74]. Similarly, a randomized controlled trial by Awakadigwe et al. reported that although active and expectant management produced comparable delivery modes and complication rates, active management significantly reduced both the latency period and the induction-to-delivery interval [75]. In the same meta-analysis, induction with vaginal prostaglandins was associated with more chorioamnionitis than oxytocin induction [74]. In the absence of contraindications, vaginal birth remains the preferred route of delivery for PROM beyond 28 weeks, as it is associated with less maternal and perinatal morbidity than caesarean delivery [76].

### Home/outpatient management of women with PROM

The role of outpatient management in women with PPROM remains incompletely defined because the supporting evidence is limited and heterogeneous. Dussaux et al. conducted a retrospective cohort study of 90 women with PPROM between 24 and 34 weeks managed at home and compared them with 324 women managed in hospital. They found that outpatient care was not associated with major maternal or neonatal complications [77]. Likewise, Carlan et al. performed a randomized controlled trial in which 67 women with PPROM were allocated to either home-based or hospital-based management, and they reported no significant differences in perinatal outcomes between the two groups [78]. However, a Cochrane review comparing home versus hospital management for PPROM at less than 37 weeks identified only two small studies, both of which were underpowered to detect clinically important differences [79]. As such, the current evidence base is insufficient to support routine outpatient care for all women with PROM.

This uncertainty is reflected in current clinical guidance. The most recent Royal College of Obstetricians and Gynaecologists (RCOG) guideline is the first major guideline to explicitly address outpatient management and recommends that the decision should be individualized. It further acknowledges that factors influencing latency to delivery should be taken into account and that the optimal monitoring strategy for predicting adverse fetal outcomes after PPROM remains uncertain [63,80]. By contrast, ACOG, in guidance published one year later, does not support outpatient care during the viable period, citing insufficient evidence regarding safety.

Even in settings where outpatient care is offered selectively, important uncertainties remain regarding the frequency, content, and effectiveness of surveillance. Hall et al. recently examined monitoring practices, clinical outcomes, and patient experience in women with PPROM managed as outpatients [81]. Their study collected retrospective data on demographic characteristics, baseline risk factors, diagnostic details, outpatient monitoring practices, maternal observations, biochemical tests, ultrasound findings, delivery outcomes, and placental histopathology [81]. Counselling practices were also evaluated both at diagnosis and during the first consultant review, and women were invited to participate in a service evaluation regarding their care experience. Across six participating units, 233 women with PPROM were managed in outpatient services. The median gestational age at diagnosis was 32 weeks, and around 34% of diagnoses were equivocal. The study found marked variation across centres in the counselling provided regarding pregnancy outcomes [81]. Spontaneous preterm birth occurred in 41% of women, while an additional 18% required preterm delivery for suspected chorioamnionitis. All participating units used maternal pulse rate, temperature, CRP, and white cell count to assess infection, but neither the absolute values at delivery nor their change over time predicted histological chorioamnionitis. Although most women considered outpatient care acceptable, many reported insufficient emotional support during management [81]. These findings suggest that outpatient care may be feasible for carefully selected women, but standardized monitoring pathways and supportive care frameworks are still needed.

### Prediction and prevention of PROM

Efforts to identify reliable predictors of PROM have generated interest in a range of biochemical and hematologic markers, although no test has yet entered routine clinical use. Underhill et al. suggested that maternal serum biglycan, decorin, and sex hormone-binding globulin (SHBG) may have potential as predictive markers of PPROM in otherwise asymptomatic women [82]. Other studies have explored additional candidate markers, including elevated second-trimester maternal serum amyloid A, increased platelet-to-lymphocyte ratio (PLR), increased neutrophil-to-lymphocyte ratio (NLR), and placental protein 14 [83–85]. While these biomarkers are of research interest, their predictive performance has not been sufficiently validated for routine clinical application.

Preventive strategies have focused primarily on addressing known risk factors. Modifiable factors include cessation of cigarette smoking, prompt treatment of urogenital, respiratory, and periodontal infections, and amnioreduction in women with polyhydramnios to decrease excessive uterine distension [6]. Management of non-modifiable or partially modifiable risk states may include vaginal progesterone and cerclage in women with a short cervix or a history of previous preterm birth [6]. Woods et al. proposed that reactive oxygen species may contribute to PROM and suggested vitamin C and E supplementation as possible preventive interventions [86]. However, higher-quality evidence has not supported the effectiveness of such approaches. In a systematic review and meta-analysis, El-Achi et al. evaluated 29 studies examining 10 interventions, including docosahexaenoic acid (DHA), aspirin, rofecoxib, vitamin C alone or combined with vitamin E, folic acid in different formulations, zinc, calcium, copper, and treatment of bacterial vaginosis [87]. They concluded that none of these interventions significantly reduced the incidence of PPROM. At present, no biomarker or preventive strategy can be recommended as a proven method for reliably predicting or preventing PROM.

### Amnioinfusion in the management of PPROM

Amnioinfusion, used to restore amniotic fluid volume, has been investigated as a potential intervention in PPROM. A Cochrane review by Hofmeyr et al found that transcervical amnioinfusion improved fetal umbilical artery pH and reduced variable decelerations, while transabdominal amnioinfusion was associated with reductions in neonatal death, sepsis, pulmonary hypoplasia, and puerperal infection [88]. However, these findings were based on a small number of trials and were largely influenced by a single study with methodological limitations, warranting cautious interpretation. Similarly, a meta-analysis by Celik et al evaluating serial transabdominal amnioinfusion in early PPROM demonstrated prolongation of latency and improved longterm survival in observational studies, although these benefits were not consistently confirmed in randomised trials [89]. Overall, current evidence remains limited and inconclusive, and routine use of amnioinfusion in PPROM cannot be recommended pending further high-quality randomised controlled trials [88, 89].

### Removal versus retention of cerclage in preterm premature rupture of membranes

Cervical cerclage is an established intervention for preventing preterm birth but is associated with complications such as PPROM and chorioamnionitis [90, 91]. The optimal timing of cerclage removal following PPROM remains debated. Evidence from a meta-analysis by Zullo et al suggests that immediate removal reduces latency but is associated with lower rates of chorioamnionitis and adverse neonatal outcomes, highlighting a trade-off between prolonging pregnancy and infection risk [90]. A pragmatic approach involves short-term retention (approximately 24 hours) to allow completion of antenatal corticosteroid therapy, followed by removal, thereby balancing neonatal benefits and infection risks [91]. However, findings across studies remain inconsistent. Current guidance, supported by Ghareeb et al, recommends expectant management with cerclage retention until 32–34 weeks’ gestation in the absence of labour, infection, or bleeding [91]. Further robust evidence is needed to guide optimal management.

### PROM and breech presentation

Evidence on term premature rupture of membranes (PROM) in breech presentation is limited, despite clinical concerns regarding cord prolapse and infection risk. Evidence on term PROM in breech presentation remains limited, despite concerns about cord prolapse and infection. Rasch et al conducted a prospective cohort study of 2876 women with singleton breech presentation, including 1920 planned vaginal deliveries (642 PROM; 1278 spontaneous rupture of membranes [SROM]) [92]. No significant differences were observed in maternal or neonatal outcomes between PROM and SROM groups, including comparable modified PREMODA scores [92]. However, PROM was associated with slightly higher maternal body mass index and increased use of epidural analgesia. It was concluded that PROM does not appear to worsen outcomes in term breech deliveries when managed by experienced clinicians, supporting management approaches similar to cephalic presentations while maintaining vigilance for cord prolapse in selected cases [92].

## Conclusion

Premature rupture of membranes (PROM) remains a significant obstetric challenge due to its contribution to preterm birth and associated neonatal morbidity and mortality. A prior history of PROM is the strongest risk factor, highlighting the importance of risk stratification. Diagnosis is primarily clinical, with adjunctive tests such as PAMG-1 and IGFBP-1 reserved for equivocal cases. Management is guided by gestational age and the balance between infection and prematurity risks, incorporating antibiotics, antenatal corticosteroids, and magnesium sulphate for fetal neuroprotection before 34 weeks. Tocolysis is not routinely recommended but may be used short term for specific indications. Expectant management is generally appropriate for preterm PROM in the absence of contraindications, while active management is preferred at term. Currently, no reliable strategies exist for the prediction or prevention of PROM. Emerging approaches, including outpatient care and amnioinfusion, remain investigational. Clinical decision-making should also consider complexities such as cerclage retention and PROM in breech presentation, while exercising caution in the interpretation of diagnostic tests in uncertain cases.

## Figures and Tables

**Table 1:** Summary of cases of premature rupture of fetal membranes (PROM)

Authors	Maternal age (years)	Parity	Gestational age (weeks)	Clinical presentation	Specific investigation findings	Treatment	Gestational age at delivery	Mode of delivery	Feto-maternal outcome
Early et al.^41^	35 years	G4P2^+1^	13 weeks plus 3 days	Leakage of fluid per vagina following CVS. VE: Positive pooling, nitrazine, and ferning test.	U/SS: Oligohydramnios.	Expectant management . Patient reported no drainage of liquor on the 3^rd^ day.	36 weeks	Elective induction of labor/vaginal delivery.	Live female neonate, that weighed 2.7kg with good APGAR score.
Hidalgo-Chicharro et al.^42^	26 years	G3P2	32 weeks	Discharge of clear fluid per vagina. VE: Closed cervix with clear fluid in the vagina.	U/SS: Viable singleton fetus with adequate liquor volume,	Conservative management : Antibiotics, steroid,	33 weeks plus 4 days	Induction of labor/vaginal delivery	Live female baby that weighed 1.8kg with good APGAR Scores. Admitted in NICU and subsequently discharged
Ekpa et al.^43^	30 years	G2P1	29weeks plus 6days	Leakage of fluid per vagina of 2 hours duration. VE: Pool of liquor in posterior vaginal fornix, liquor trickling down an open cervical os on valsalva maneuver	U/SS: Viable pregnancy with severe oligohydramnios (AFI = 0.8cm)	Conservative management : Antibiotics, tocolysis, steroid, bed rest,	30 weeks plus 2 days.	Emergency caesarean section.	Live 1.4kg female neonate.with good APGAR score. Baby was subsequently discharged after two weeks from the SCBU. Mother had pneumonia and was treated appropriately and discharged
Alkasseh et al.^44^	32 years	G4P3	32weeks	Discharge of clear fluid per vagina. VE: Speculum shows pool of liquor in the posterior fornix,	U/SS: Closed cervix, adequate liquor,	Conservative management : Antibiotics, steroid, tocolysis.	33 weeksplus 4 days.	Vaginal delivery following induction of labor.	Live female neonate that weighed 1.97kg with good APGAR score. Subsequently discharged on 13^th^ day post-delivery from NICU.
Jha et al.^45^	25 years	Primigravida	20 weeks	Drainage of liquor per vagina. VE: Speculum shows pool on liquor in the vagina.	U/SS: AFI of 9.4cm and 3.2cm on first and fourth day.	Expectant management : Antibiotics, pelvic rest, Subsequently discharged 10^th^ day on admission following cessation of liquor.	37 weeks	Caesarean delivery	Live 3.2kgmale baby with satisfactory APGAR score. Mother was stable.
Shaheed et al.^46^	Not mentioned	Primigravida	28	Watery vaginal discharge of 2 weeks duration. VE: closedcervix, liquor trickling down the cervix.	U/SS: Viable singleton pregnancy at 31+ weeks with moderate oligohydraminous.	Antibiotics, hormonal support, and steroid	32 weeks	Emergency caesarean section	Healthy, live 2.7kg female baby with good APGAR score. Baby subsequently discharged from NICU following phototherapy on account of neonatal jaundice.
Chmaj-Wierzchowska et al.^47^	27	Primipara	19weeks	Profuse vaginal discharge x 2 weeks. VE: Closed cervix, subsequent speculum examination revealed pool of amniotic fluid in the vagina	U/ss: Normal for gestational age fetus, oligohydramnios, elevated CRP, positive vaginal culture	Initial intravaginal suppository antibiotics, Magnesium hydoaspartate, drotaverine hydrochloride, Betamethasone,	31/32weeks	Caesarean section	Live 1.2kg male baby. Discharged subsequently in good condition
Wu et al.^48^	32 years	Primigravida	18 weeks	Drainage of fluid per vagina following amniocentesis on account of positive maternal serum screening for down syndrome in a twin pregnancy.	U/SS: Nearly no liquor for twin B.	Antibiotics, tocolytics	30 weeks plus 2days	Emergency Caesarean section due to abruptio placenta	Live twin babies that weighed 1.5kg (Twin A) and 0.83kg (Twin B). Both twin had PDA, received ventilation support. Twin B had severe IUGR and IVH, and subsequently died.

VE = Vaginal examination, U/SS = Ultrasonography, IVH = Intra-ventricular haemorrhage, IUGR = Intra-uterine growth restriction, PDA = Patent ductus arteriosus, AFI = Amniotic fluid index, NICU = Neonatal intensive care unit

## Data Availability

Not applicable

## Abbreviations:

PROM: Premature Rupture of foetal Membranes
PPROM: Preterm PROM
PTB: Preterm Birth
IVH: Intraventricular Haemorrhage
IUGR: Intrauterine Growth Restriction
PDF: Patent Ductus Arteriosus
AFI: Amniotic Fluid Index
NICU: Neonatal Intensive Care Unit
NAUTH: Nnamdi Azikiwe University Teachng Hospital
RR: Relative Risk
CI: Confidence Interval
BMI: Body Mass Index
IPI: Inter Pregnancy Interval
GDM: Gestational Diabetes Mellitus
DHA: Docasahexaenoic Acd
IL-1β: Interleukin 1 beta
TNF-α: Tumour necrosis factor alpha
ALPS: Antenatal Late Preterm Steroids
RCT: Randomized Controlled Trial
RDS: Respiratory Distress Syndrom
SHBG: Sex Hormone Binding Globulin
PLR: Platelet-to-lymphocyte ratio
NLR: Neutrophil-to-lymphocyte ratio
NICE: National Institute for Health and Care Excellence
ORACLE: Overview of the Role of Antibiotics in Curtailing Labour and Early delivery
ACOG: American College of Obstetricians and Gynaecologists
SOGC: Society of Obstetricians and Gynaecologists of Canada
WHO: World Health Organisation
IL-6: Interleukin 6
MMP: Matrix Metalloproteinases
PAMG-1: Placental alpha microglobulin-1
IGFBP-1: Insulin-like growth factor binding protein-1
AFP: Alpha-fetoprotein
CRP: C-reactive protein

## References

[1] Delorme P, Lorthe E, Sibiude J, Kayem G. (2021). Preterm and term prelabour rupture of membranes: A review of timing and methods of labour induction. Best Pract Res Clin Obstet Gynaecol. 77:27–41. 34538740 10.1016/j.bpobgyn.2021.08.009

[2] Monson MA, Gibbons KJ, Esplin MS, Varner MW, Manuck TA. (2016). Pregnancy outcomes in women with a history of previable, preterm prelabor rupture of membranes. Obstet Gynecol. 128(5):976–982. 27741176 10.1097/AOG.0000000000001682 PMC5774863

[3] Ubom AE, Vatish M, Barnea ER. (2023). FIGO Childbirth and Postpartum Hemorrhage Committee. FIGO good practice recommendations for preterm labor and preterm prelabor rupture of membranes: prep-for-labor triage to minimize risks and maximize favorable outcomes. Int J Gynecol Obstet. 163:40–50. 10.1002/ijgo.15113 37807588

[4] Wang X, Zhang X, Liu Y, Jiang T, Dai Y. (2020). Effect of premature rupture of membranes on time to delivery and outcomes in full-term pregnancies with vaginal dinoprostone-induced labour. Arch Gynecol Obstet. 301:369–374. 31776709 10.1007/s00404-019-05351-1

[5] Eleje GU, Ezebialu IU, Umeobika JC, Eke AC, Ezeama CO. (2010). Prelabour rupture of membranes at term: a review of management in a health care institution. Afrimedic J. 1(2):10–14.

[6] Waters TP, Mercer B. (2011). Preterm PROM: prediction, prevention, principles. Clin Obstet Gynecol. 54(2):307–12. 21508700 10.1097/GRF.0b013e318217d4d3

[7] Aris IM, Logan S, Lim C, Choolani M, Biswas A, Bhattacharya S.(2017). Preterm prelabour rupture of membranes: a retrospective cohort study of association with adverse outcome in subsequent pregnancy. BJOG 124(11):1698–707. 28029224 10.1111/1471-0528.14462

[8] Mercer BM, Goldenberg RL, Meis PJ, Moawad AH, Shellhaas C, Das A, (2000). The preterm prediction study: prediction of preterm premature rupture of the membranes using clinical findings and ancillary testing. Am J Obstet Gynecol. 183:738–745. 10992202 10.1067/mob.2000.106766

[9] Mercer BM, Goldenberg RL, Moawad AH, Meis PJ, Iams JD, (1999).The preterm prediction study: effect of gestational age and cause of preterm birth on subsequent obstetric outcome. Am J Obstet Gynecol. 181:1216–1221. 10561648 10.1016/s0002-9378(99)70111-0

[10] Emechebe CI, Njoku CO, Anachuna K, Udofia U. (2015). Determinants and complications of pre-labour rupture of membranes (PROM) at the University of Calabar Teaching Hospital (UCTH), Calabar, Nigeria. Sch J App Med Sci. 3(5B):1912–1917.

[11] Tchirikov M, Schlabritz-Loutsevitch N, Maher J, Buchmann J, Naberezhnev Y, (2018). Mid-trimester preterm premature rupture of membranes (PPROM): etiology, diagnosis, classification, international recommendations of treatment options and outcome. J Perinat. 46(5):465–488. 10.1515/jpm-2017-0027 28710882

[12] Lin D, Hu B, Xiu Y, Ji R, Zeng H, (2024). Risk factors for premature rupture of membranes in pregnant women: a systematic review and meta-analysis. BMJ Open. 14(3):e077727. 10.1136/bmjopen-2023-077727 PMC10982755 38553068

[13] Maymon E, Romero R, Pacora P, Gervasi MT, Bianco K. (2000). Evidence for the participation of interstitial collagenase (matrix metalloproteinase 1) in preterm premature of membranes. Am J Obstet Gynecol. 183:914–20. 11035337 10.1067/mob.2000.108879

[14] Maymon E, Romero R, Pacora P, Gomez R, Athayde N. (2000). Human neutrophil collagenase (matrix metalloproteinase 8) in parturition, premature rupture of the membranes, and intrauterine infection. Am J Obstet Gynecol. 183:94–99. 10920315 10.1067/mob.2000.105344

[15] Athayde N, Edwin SS, Romero r, Gomez R, Maymon E. (1998).A role for matrix metalloproteinase-9 in spontaneous rupture of the fetal membranes. Am J Obstet Gynecol 179:1248–1253. 9822510 10.1016/s0002-9378(98)70141-3

[16] Maymon E, Romero R, Pacora R, Gervasi MT, Gomez R. (2000). Evidence of in vivo differential bioavailability of the active forms of matrix metalloproteinases 9 and 2 in parturition, spontaneous rupture of membranes, and intra-amniotic infection. Am J Obstet Gynecol 183:887–894. 11035332 10.1067/mob.2000.108878

[17] Eleje GU, Adinma JI, Ugwuanyi DC, Ikechebelu JI, Okafor CI. (2015). Genital tract microbial isolate in women with preterm prelabour rupture of membranes in resource-constrained community setting. J Obstet Gynaecol. 35(5):465–468. 25358030 10.3109/01443615.2014.970145

[18] Temur I, Temur KT, Donertas SN, Dönertas AD. (2024).The relationships of inflammatory blood markers with maternal periodontal and dental states and their effects on preterm membrane rupture development. BMC Oral Health. 24(1):652. 38835011 10.1186/s12903-024-04427-y PMC11149273

[19] Garite TJ. (2001).Management of prematurerupture of membranes. Clin Perinatol. 28: 837–847. 11817193 10.1016/s0095-5108(03)00081-2

[20] American College of Obstetricians and Gynecologists’ Committee on Practice Bulletins—Obstetrics. Practice bulletin no. 172: premature rupture of membranes. Obstet Gynecol. 2016;128:e165–e177. 27661655 10.1097/AOG.0000000000001712

[21] Alexander JM, Mercer BM, Miodovnik M, Thurnau GR, Goldenberg RL. (2000).The impact of digital cervical examination on expectantly managed preterm rupture of membranes. Am J Obstet Gynecol. 183:1003–1007. 11035354 10.1067/mob.2000.106765

[22] Ayyar A, Moufarrij S, Turrentine M. (2022). Infectious morbidity of speculum versus digital examinations in preterm prelabor rupture of membranes: a systematic review and meta-analysis. J Matern.-Fetal Neonatal Med. 35(25):8905–11. 34818968 10.1080/14767058.2021.2006628

[23] Munson LA, Graham A, Koos BJ, Valenzuela GJ. (1985).Is there a need for digital examination in patients with spontaneous rupture of the membranes? Am J Obstet Gynecol. 153(5):562–563. 4061518 10.1016/0002-9378(85)90474-0

[24] Deshpande SN, van Asselt ADI, Tomini F, Armstrong N, Allen A. (2013). Rapid fetal fibronectin testing to predict preterm birth in women with symptoms of premature labour: A systematic review and cost analysis. 40 ed. NIHR Journals Library, 138 p. (Health Technology Assessment). 10.3310/hta17400 PMC4781038 24060096

[25] Van Der Ham DP, Van Melick MJ, Smits L, Nijhuis JG, Weiner CP. (2011). Methods for the diagnosis of rupture of the fetal membranes in equivocal cases: a systematic review. Eur J Obstet Gynecol Reprod Biol. 157(2):1237. 10.1016/j.ejogrb.2011.03.006 21482018

[26] Medina TM, Hill DA. (2006). Preterm premature rupture of membranes: diagnosis and management. Am Fam Physician. 73:659–664. 16506709

[27] Lee SE, Park JS, Norwitz ER, Kim KW, Park HS, Jun JK. (2007).Measurement of placental alpha-microglobulin-1 in cervicovaginal discharge to diagnose rupture of membranes. Obstet Gynecol. 109:634–640. 17329514 10.1097/01.AOG.0000252706.46734.0a

[28] Eleje GU, Ezugwu EC, Ogunyemi D, Eleje LI, Ikechebelu JI. (2015).Accuracy and cost‐analysis of placental alpha‐ microglobulin‐1 test in the diagnosis of premature rupture of fetal membranes in resource‐limited community settings. J Obstet Gynaecol Res.. 41(1):29–38. 25164109 10.1111/jog.12475

[29] Abdelazim IA, Shikanova S, Karimova B, Sarsembayev M, Mukhambetalyeva G, (2020). Diagnostic accuracy of insulin-like growth factor–binding protein1/alpha-fetoprotein (amnioquick duo) in ruptured fetal membranes. SN Compr Clin Med. 2(12):2834–2839.

[30] Eleje GU, Ezugwu EC, Eke AC, Ikechebelu JI, Ezeama CO. (2017).Accuracy and response time of dual biomarker model of insulin‐like growth factor binding protein‐1/alpha fetoprotein (Amnioquick duo+) in comparison to placental alpha‐microglobulin‐ 1 test in diagnosis of premature rupture of membranes. J Obstet Gynaecol Res. 43(5):825–833. 28422393 10.1111/jog.13296

[31] Palacio M, Kühnert M, Berger R, Larios CL, Marcellin L. (2014). Meta-analysis of studies on biochemical marker tests for the diagnosis of premature rupture of membranes: comparison of performance indexes. BMC Pregnancy Childbirth. 14:1–2. 24884494 10.1186/1471-2393-14-183 PMC4229884

[32] National Institute for Health and Care Excellence. Preterm Labour and Birth. NICE guideline [NG25]. NICE; 2015. 37184164

[33] Kong X, Jiang L, Zhang B, Sun L, Liu K. (2023). Predicting chorioamnionitis in patients with preterm premature rupture of membranes using inflammatory indexes: a retrospective study. Taiwan J Obstet Gynecol. 62(1):112–118. 36720521 10.1016/j.tjog.2022.11.006

[34] Beydoun SN, Yasin SY. (1986). Premature rupture of the membranes before 28 weeks: conservative management. Am J Obstet Gynecol. 155:471–479. 3752169 10.1016/0002-9378(86)90257-7

[35] Tita ATN, Andrews WW. (2010).Diagnosis and management of clinical chorioamnionitis. Clin Perinatol. 37:339–354. 20569811 10.1016/j.clp.2010.02.003 PMC3008318

[36] Lewis DF, Adair CD, Weeks JW, Barrilleaux PS, Edwards MS. (2000). A randomized clinical trial of daily nonstress testing versus biophysical profile in the management of preterm premature rupture of membranes. Am J Obstet Gynecol. 183:777–778. 10601934 10.1016/s0002-9378(99)70395-9

[37] Caloone J, Rabilloud M, Boutitie F, Traverse-Glehen A, Allias-Montmayeur F. (2016). Accuracy of several maternal seric markers for predicting histological chorioamnionitis after preterm premature rupture of membranes: a prospective and multicentric study. Eur J Obstet Gynecol Reprod Biol. 205:133–140. 27591714 10.1016/j.ejogrb.2016.08.022

[38] Sabogal CP, Fonseca J, García-Perdomo HA. (2018). Validation of diagnostic tests for histologic chorioamnionitis: systematic review and meta-analysis. Eur J Obstet Gynecol Reprod Biol. 228:13–26. 29908373 10.1016/j.ejogrb.2018.05.043

[39] Eleje GU, Ukah CO, Onyiaorah IV, Ezugwu EC, Ugwu EO. (2020). Diagnostic value of Chorioquick for detectingchorioamnionitis in women with premature rupture of membranes. Int J Gynecol Obstet. 149(1):98–105. 10.1002/ijgo.13095 31907923

[40] Etyang AK, Omuse G, Mukaindo AM, Temmerman M. (2020).Maternal inflammatory markers for chorioamnionitis in preterm prelabour rupture of membranes: a systematic review and meta-analysis of diagnostic test accuracy studies. Syst Rev. 9:1–4. 32532314 10.1186/s13643-020-01389-4 PMC7293113

[41] Early J, Arrabal P. (2023). Preterm prelabor rupture of membranes after first-trimester chorionic villus sampling: A case report and review of the literature. Case Rep Womens Health. 18;41:e00577. 10.1016/j.crwh.2023.e00577 PMC10788396 38226353

[42] Hidalgo-Chicharro A, Abad-Torreblanca R, Navarro-Marí JM, Gutiérrez-Fernández J. (2017). 32-week premature rupture of membranes caused by oropharyngeal microbiota. JMM Case Rep. 4:e005121. 29188068 10.1099/jmmcr.0.005121 PMC5692237

[43] Ekpa QL, Udoudo MI, Nwebeh EI, Nwebeh OC. (2024). Preterm Prelabour Rupture of Membrane (PPROM) in a Young Female in South-South Nigeria: A Clinical Case Report. Cureus. 16(1):e51649. 38313973 10.7759/cureus.51649 PMC10838058

[44] Alkasseh AS, Alkhatib SJ. (2021). A Case Study On 3 Weeks Premature Rupture Of Membranes Caused By Oropharyngeal Microbiota. Innovare Journal of Health Sciences. 19:1–2.

[45] Jha A, Li X, Zhang S. and Li H. (2019). Successful Management of Preterm Premature Rupture of Membrane in Second Trimester: A Case Report and Literature Review. Yangtze Medicine. 3:149–156.

[46] Shaheed Saika, Haque Munima, and Haider Rebeka. (2019). “Successful Management of Prematured Rupture of Membrane (PROM): A Case Report.”

[47] Chmaj-Wierzchowska K, Pięta B, Buks J, (2012). Determinants of favourable neonatal outcome after premature rupture of membranes (PROM) before 24 weeks of pregnancy-review of the literature and a case report. Ann Agric Environ Med. 19(3): 577–580. 23020060

[48] Wu MY, Chen SU, Lee CN, Ho HN, Yang YS. (2010). Use of atosiban in a twin pregnancy with extremely preterm premature rupture in the membrane of one twin: a case report and literature review. Taiwan J Obstet Gynecol. 49(4):495–499. 21199753 10.1016/S1028-4559(10)60103-9

[49] Kenyon S, Boulvain M, Neilson JP. (2013). Antibiotics for preterm rupture of membranes. Cochrane Database Syst Rev. 12:CD001058. 10.1002/14651858.CD001058 12804398

[50] Kenyon SL, Taylor DJ, Tarnow-Mordi W. (2001). Broad-spectrum antibiotics for preterm, prelabour rupture of fetal membranes: the ORACLE I randomised trial. Lancet. 357:979–988. 11293640 10.1016/s0140-6736(00)04233-1

[51] Mercer BM, Miodovnik M, Thurnau GR, Goldenberg RL, Das AF. (1997). Antibiotic therapy for reduction of infant morbidity after preterm premature rupture of the membranes. A randomized controlled trial. National Institute of Child Health and Human Development Maternal–Fetal Medicine Units Network. JAMA 278:989–995. 9307346

[52] Yudin MH, van Schalkwyk J, Van Eyk N. (2017). No. 203-antibiotic therapy in preterm premature rupture of the membranes. J Obstet Gynaecol Can. 39:e207e212. 28859768 10.1016/j.jogc.2017.06.003

[53] Magann EF, Haram K, Ounpraseuth S, Mortensen JH, Spencer HJ. (2017).Use of corticosteroids in special circumstances: a comprehensive review. Acta Obstet Gynecol Scand 96:395–409. 28130929 10.1111/aogs.13104

[54] (2020). WHO Action Trials Collaborators. Antenatal dexamethasone for early preterm birth in low resource countries. N Engl J Med. 383(26):2514–25. 33095526 10.1056/NEJMoa2022398 PMC7660991

[55] Brownfoot FC, Gagliardi DI, Bain E, Middleton P, Crowther CA. (2013). Different corticosteroids and regimens for accelerating fetal lung maturation for women at risk of preterm birth. Cochrane Database Syst Rev. 8:CD006764. 10.1002/14651858.CD006764.pub3 23990333

[56] (2002).ACOG committee opinion. Antenatal corticosteroid therapy for fetal maturation. Int J Gynaecol Obstet. 78(1):95–97. 12197491

[57] Gyamfi-Bannerman C, Thom EA, Blackwell SC, (2016). Antenatal betamethasone for women at risk for late preterm delivery. N Engl J Med. 374:1311–1320. 26842679 10.1056/NEJMoa1516783 PMC4823164

[58] Doyle LW, Crowther CA, Middleton P, Marret S, Rouse D. (2009). Magnesium sulphate for women at risk of preterm birth for neuroprotection of the fetus. Cochrane Database Syst Rev.(1):CD004661. 19160238 10.1002/14651858.CD004661.pub3

[59] Bain E, Middleton P, Crowther CA. (2012). Different magnesium sulphate regimens for neuroprotection of the fetus for women at risk of preterm birth. Cochrane Database Syst Rev. (2):CD009302. 22336863 10.1002/14651858.CD009302.pub2 PMC11472847

[60] Magee LA, De Silva DA, Sawchuck D, Synnes A, von Dadelszen P. (2019). No. 376magnesium sulphate for fetal neuroprotection. J Obstet Gynaecol Can. 41:505–522. 30879485 10.1016/j.jogc.2018.09.018

[61] Shennan A, Suff N, Jacobsson B, (2021).FIGO Working Group for Preterm Birth. FIGO good practice recommendations on magnesium sulfate administration for preterm fetal neuroprotection. Int J Gynecol Obstet. 155:31–33.

[62] Mackeen AD, Seibel-Seamon J, Muhammad J, Baxter JK, Berghella V. (2014). (Tocolytics for preterm premature rupture of membranes. Cochrane Database Syst Rev. 2):CD007062. 24578236 10.1002/14651858.CD007062.pub3 PMC11194776

[63] Thompson AJ, (2019). Royal College of Obstetricians and Gynaecologists. Care of women presenting with suspected preterm prelabour rupture of membranes from 24+0 weeks of gestation. BJOG. 126:e152–e166. 31207667 10.1111/1471-0528.15803

[64] van Vliet EO, Nijman TA, Schuit E, (2016). Nifedipine versus atosiban for threatened preterm birth (APOSTELIII): a multicentre, randomised controlled trial. Lancet. 387:2117–2124. 26944026 10.1016/S0140-6736(16)00548-1

[65] (2015).World Health Organization. Recommendations on Interventions to Improve Preterm Birth Outcomes. WHO; 26447264

[66] Doret M, Kayem G. (2016).Tocolysis for preterm labor without premature preterm rupture of membranes. J Gynecol Obstet Biol Reprod. 45:1374–1398. 10.1016/j.jgyn.2016.09.018 28029463

[67] Sorrenti S, Di Mascio D, Khalil A, D’Antonio F, Rizzo G. (2024). Outcome of prelabour rupture of membranes before or at the limit of viability: systematic review and meta-analysis. Am J Obst Gynec MFM. 6(6):101370. 38648897 10.1016/j.ajogmf.2024.101370

[68] Sim WH, Ng H, Sheehan P. (2020).Maternal and neonatal outcomes following expectant management of preterm prelabor rupture of membranes before viability. J Matern-Fetal Neonatal Med. 33(4):533–541. 29961407 10.1080/14767058.2018.1495706

[69] Fernandes GL, Torloni MR, Hisaba WJ, Klimke D, Novaes J. (2012). Premature rupture of membranes before 28 weeks managed expectantly: maternal and perinatal outcomes in a developing country. J Obstet Gynaecol. 32(1):45–49. 22185536 10.3109/01443615.2011.609923

[70] Al-Mandeel H, Alhindi MY, Sauve R (2012). Effects of intentional delivery on maternal and neonatal outcomes in pregnancies with preterm prelabour rupture of membranes between 28 and 34 weeks of gestation: a systematic review and metaanalysis. J Matern-Fetal Neonatal Med. 26(1);83–89. 22882130 10.3109/14767058.2012.718388

[71] Royal College of Obstetricians & Gynaecologists. Preterm Prelabour Rupture of Membranes Green–Top Guideline No. 44 November 2006.

[72] Bond DM, Middleton P, Levett KM, van der Ham DP, Crowther CA. (2017). Planned early birth versus expectant management for women with preterm prelabour rupture of membranes prior to 37 weeks’ gestation for improving pregnancy outcome. Cochrane Database of Syst Rev CD004735. 28257562 10.1002/14651858.CD004735.pub4 PMC6464692

[73] van der Heyden JL, Willekes C, van Baar AL, van Wassenaer-Leemhuis AG, Pajkrt E, (2015). Behavioural and neurodevelopmental outcome of 2-year-old children after preterm premature rupture of membranes: follow-up of a randomised clinical trial comparing induction of labour and expectant management. Eur J Obstet Gynecol Reprod Biol 194:17e23. 26319651 10.1016/j.ejogrb.2015.07.014

[74] Mozurkewich EL, Wolf FM. (1997). Premature rupture of membranes at term: a metaanalysis of three management schemes. Obstet Gynecol. 89(6):1035–1043. 9170488 10.1016/s0029-7844(97)00094-x

[75] Awkadigwe FI, Ezugwu FO, Eleje GU, Nweze SO, Odugu BU. (2023). Active versus expectant management for premature rupture of membranes at term: A randomized, controlled study. J Int Med Res. 51(8):03000605231195451. 10.1177/03000605231195451 PMC10478565 37656970

[76] Kayiga H, Lester F, Amuge PM, Byamugisha J, Autry AM. (2018).Impact of mode of delivery on pregnancy outcomes in women with premature rupture of membranes after 28 weeks of gestation in a low-resource setting: A prospective cohort study. PloS one. 13(1):e0190388. 29320516 10.1371/journal.pone.0190388 PMC5761877

[77] Dussaux C, Senat MV, Bouchghoul H, Benachi A, Mandelbrot L, Kayem G. (2018). Preterm premature rupture of membranes: is home care acceptable? J Matern Fetal Neonatal Med. 31:2284–2292. 28612662 10.1080/14767058.2017.1341482

[78] Carlan SJ, O’Brien WF, Parsons MT, Lense JJ. (1993). Preterm premature rupture of membranes: a randomized study of home versus hospital management. Obstet Gynecol. 81:61–64. 8416463

[79] 7Abou El Senoun G, Dowswell T, Mousa HA. (2010). Planned home versus hospital care for preterm prelabour rupture of the membranes (PPROM) prior to 37 weeks’ gestation. Cochrane Database Syst Rev. (4):CD008053. 20393965 10.1002/14651858.CD008053.pub2 PMC4170988

[80] Carroll SGM on behalf of the Royal College of Obstetricians and Gynaecologists. Green Top Guideline Number 44: Preterm Prelabour Rupture of Membranes. 2010.

[81] Hall M, Dias M, Clark E, Stirrat L, Goodfellow L. (2026). Outpatient management of women with preterm prelabour rupture of the membranes: A retrospective multicentre cohort study. Eur J Obstet Gynecol Reprod Biol. 320:115016. 41713153 10.1016/j.ejogrb.2026.115016

[82] Underhill LA, Avalos N, Tucker R, Zhang Z, Messerlian G, Lechner B. (2020).Serum decorin and biglycan as potential biomarkers to predict PPROM in early gestation. Reprod Sci. 27:1620–1626. 32436194 10.1007/s43032-020-00192-9 PMC7539850

[83] Köseoğlu SB, Guzel AI, Deveer R, Tokmak A, Engin-Ustun Y. (2014). Maternal serum amyloid A levels in pregnancies complicated with preterm prelabour rupture of membranes. Ginekol Pol. 85(7):516–520. 25118503 10.17772/gp/1763

[84] Toprak E, Bozkurt M, Çakmak BD, Özçimen EE, Silahlı M. (2017).Platelet-to-lymphocyte ratio: A new inflammatory marker for the diagnosis of preterm premature rupture of membranes. J Turk Ger Gynecol Assoc. 18(3):122. 28890425 10.4274/jtgga.2017.0028 PMC5590207

[85] Wang Y, Luo H, Che G, Li Y, Gao J. (2018). Placental protein 14 as a potential biomarker for diagnosis of preterm premature rupture of membranes. Mol Med Rep. 18(1):113–122. 29749501 10.3892/mmr.2018.8967 PMC6059659

[86] Woods JR Jr, Plessinger MA, Miller RK. (2001). Vitamins C andE:missinglinksin preventing preterm premature rupture of the membranes? Am J Obstet Gynecol. 185:5–10. 11483896 10.1067/mob.2001.115868

[87] El-Achi V, Aggarwal S, Hyett J. (2022). Interventions for the Prevention of Preterm Premature Rupture of Membranes: A Systematic Review and Meta-Analysis. Fetal Diagn Ther. 49(5-6):273–278. 35772387 10.1159/000525655

[88] Hofmeyr GJ, Eke AC, Lawrie TA. (2014). Amnioinfusion for third trimester preterm premature rupture of membranes. Cochrane Database Syst Rev. 2014(3):CD000942. 24683009 10.1002/14651858.CD000942.pub3 PMC7061243

[89] Celik E, Yildiz AB, Guler Cekic S, Unal C, Ayhan I. (2023). Amnioinfusion vs. standard management for the second trimester PPROM: a systematic review and meta-analysis of observational studies and RCTs. J Matern Fetal Neonatal Med. 36(2):2230511. 37408113 10.1080/14767058.2023.2230511

[90] Zullo F, Di Mascio D. (2025). Management of cervical cerclage after preterm premature rupture of membranes: an argument for removal. Am J Obstet Gynecol MFM. 7(1S):101570. 39586472 10.1016/j.ajogmf.2024.101570

[91] Ghareeb AA, Kachikis A, Nguyen V, Roman A. (2025). Management of cervical cerclage after preterm premature rupture of membranes: an argument for retention. Am J Obstet Gynecol MFM. 7(1S):101569. 39586469 10.1016/j.ajogmf.2024.101569

[92] Rasch E, Hentrich AE, Kämpf AK, Deuster E, Hoock SC. (2026). Influence of premature rupture of membranes on the peripartum outcomes in vaginally intended breech deliveries at term: A FRABAT study. Int J Gynaecol Obstet. 172(1):630–638. 40698882 10.1002/ijgo.70391 PMC12724039

